# Nexus between social responsibilities of young cinematic celebrities and public recognition: Evidence from China

**DOI:** 10.3389/fpsyg.2022.945634

**Published:** 2022-08-24

**Authors:** Yun Zhao, Tina Eyraud, Yaowen Xue, Muhammad Imran, Kai Wang, Xiaotong Sun

**Affiliations:** ^1^Department of Economics and Management, Yuncheng University, Yuncheng, China; ^2^Department of Sociology, Northern Arizona University, Flagstaff, AZ, United States; ^3^Information Research Institute, Qilu University of Technology, Shandong Academy of Sciences, Jinan, China; ^4^School of Economics and Management, Hainan University, Haikou, China; ^5^Department of Economics and Management, Qilu University of Technology, Jinan, China

**Keywords:** cinematic celebrities, social responsibility, public recognition, SRESI, TV stars

## Abstract

As public figures, cinematic celebrities’ behaviors have widespread influence on the whole society, and this also applies to China. Their influence is reflected in public recognition. This research classifies celebrity social responsibility as behavior within the law, professionalism, family ethics, public morality, and charity. We selected 286 cinematic celebrities as study objects and obtained public recognition data through 2,600 questionnaire surveys. The findings of the study indicate that there is a positive, linear relationship between cinematic celebrities’ social responsibility and public recognition. In particular, family ethics and public moral responsibility demonstrate a significant positive correlation with public recognition. However, there was no significant correlation found between lawful responsibility and public recognition. Finally, this paper makes additional suggestions and recommendations drawn from the data and reported in the conclusion.

## Introduction

In China, particularly in recent years, cinematic celebrities have engaged in a variety of misconduct that includes, but is not limited to, endorsement of false advertising, organized criminal behavior, marital infidelity, drunk driving, fighting, and drug abuse (Ren, 2014; Zhang, 2020). The frequency of celebrity endorsement in China ranks third in the world. Some advertisements endorsed by movie stars manifest abnormal behavior, such as product distortion, false promises, and value-oriented deviation, which harm the interests of consumers and destroy the moral value of society (Liu, 2020). Based on the positive behavior evaluation indexes, movie stars’ responsibility performance scores are very low in China (Zhang and Xue, 2021). With the exposure of large-scale tax evasion (made public in June 2018) and adverse legal judgments amounting to thousands of millions Yuan in administrative punishments, cinematic celebrity social responsibility behaviors have become the focus of social attention and concern.

The subject of cinematic celebrities is very hot in marketing. Therefore, an extensive body of literature shows that celebrity endorsements lead to higher levels of consumer–brand patronage intentions. Sports apparel brands choose athletes as celebrity endorsers to enhance positive attitudes and encourage merchandise sales (Intravia et al., 2020). In sports literature, studies show that consumers are more likely to display higher levels of purchase intention when they associate themselves with endorsed athletes (Eyada, 2020). Specifically, researchers found that companies could achieve a variety of benefits from athlete endorsements, such as a consumer’s increased probability of brand choice, intention to pay a premium price, and positive word of mouth (Carrillat et al., 2019). This research investigate that how the celebrities’ reputation can be used for better social responsibilities.

As public figures, movie stars expect public recognition and so they try to present their “good moral behaviors” (their good said, as it were) in public (Guo, 2015). The audiences’ recognition of stars comes from their screen images and life images. From a societal perspective, movie stars can provide followers with value guidance, so that they should themselves be encouraged to consider their behavior in connection with their social responsibility (Wang and Yan, 2019; Stojanovic et al., 2020). However, due to some audiences’ (especially teenagers’) irrational pursuit of stars, stars demonstrating poor moral behavior may also be recognized by the public, while, conversely, stars showing good moral behavior may not attract enough recognition (Qin and Chen, 2021).

This research has two main objectives. First, it investigates the correlation between a celebrity’s social responsibility performance and public recognition. Secondly, it asks what kind of responsibility exerts significant influence on that public recognition. In order to investigate these two objectives, we selected 286 cinematic celebrities as study objects and derived their responsibilities’ scores by using an evaluation index system. Additionally, we obtained public recognition data through 2,600 questionnaire surveys and studied the relationship between cinematic celebrities’ responsibilities and public recognition. This research focuses on how to evaluate individual responsibility and the relationship between celebrities’ social responsibility performance and public recognition, and accordingly it offers suggestions to guide and encourage movie stars to be more responsible toward their society that are presented in the conclusion of the paper.

## Literature review

### Studies on the “star system”

In developed countries, research conducted on cinematic celebrities generally focuses on the “star system” and it is yet to demonstrate a clear interest in celebrity social responsibility consciousness and behavior relative to their communities and to society at large. Instead, research emphasis is concentrated on systemic production of star imagery.

The “star system” originated with the American film industry (Dyer, 1979). It was, and remains, a mechanism of propaganda and promotion for commercial operations to construct a specific symbolic image (personality, identity, and appearance) of a celebrity to be disseminated to the general public. The “star system” was described as an operation mode by Thomas Harris. Under that mode, celebrities are subservient to an interest group (Harris, 1991). Charles Eckert’s “Jump Cut” in 1974 refers to the philanthropic-star image created purposefully during the Depression to mobilize the wealthy classes to bear the financial burdens of that era (Eckert, 1974). This mature “star system” mechanism has built the star image that has, to a very considerable extent, been accepted by the public, and it maintained as the star’s public image. Under this mechanism, the star is created by complicated production procedures. The star is subject to economic power and autocracy, by which their public image is manipulated and consolidated.

Therefore, in western countries, under the “star system’s” mature operation mode, the individual star is subject to the design and arrangement of the overall system, an amalgamation of the economic needs of the film industry and the acceptance of the general public. The social responsibility of movie stars themselves does not appear as a prominent social problem, and there is little related research on that.

### Research on celebrity social responsibility

There are many studies about social responsibility, but these tend to concentrate on the social responsibility of enterprises, not of individuals (Zhong, 2018; Shimul et al., 2020). Some related research has noted celebrity misconduct, such as advertising endorsements, drug abuse, and divorce, but few studies have defined the concept of (individual) celebrity social responsibility. Researchers have begun to distinguish corporate social responsibility (CSR) and corporate social advocacy (CSA). Because CSA can often promote challenging beliefs and objectives, it is frequently distinguished from CSR, which entails charitable support for broadly popular projects. Because the nature of the corporate support is different, CSR and CSA efforts generate distinctive consumer responses. While CSA often results in polarized reactions, CSR messages usually evoke support or general ambivalence from customers (Rim et al., 2020).

Celebrity social obligations include economic, political, cultural, and moral responsibility (Lin, 2002; Hao and Zhang, 2009). In addition, stars are expected to fulfill their philanthropic responsibility, especially with charitable contributions to promote the moral growth of youth and improve the level of civilized behavior both in local communities and the whole society. The premise of fulfilling responsibilities by stars is that they have a moral responsibility and should play a role in demonstration of moral behavior to the public (Yu, 2007). In this way, celebrity fulfillment of social responsibility is an ethical requirement. Accepting that responsibility meets stated ethical requirements, whereas shirking or refusing to do so is contrary to those requirements (Li, 2011).

Negative celebrity behaviors can be divided into two major categories: illegal behaviors and immoral behaviors (Zhang and Zhao, 2016). There are many studies focusing on advertising endorsement from a legal standpoint. A star who has provided a false endorsement must bear a corresponding legal liability (Yao and Duan, 2009; Wu, 2010). At the very least, stars who endorse products must bear prudential and self-certification obligations (Liu N. L., 2015).

Also from a legal standpoint, research on drug abuse incidents involving public figures indicates that social responsibility includes two levels. The first is the level of aggregation, which is the lawful requirement of individual stars. The second is the level of discrete meaning, referring directly to mandatory obligation (Liu and Wu, 2015). In China, large-scale tax evasion by celebrities have been demarcated as breaking the “bottom line” of the law, which means that celebrities, collectively, may be seen as lacking in their sense of legal responsibility and are thereby acting against basic social justice (Zhong, 2018). Liu explains this phenomenon as a consequence attributed to factors such as disorder in the moral value system and the decline of an individual’s conscious morality, particularly during periods of social transition (Liu Y., 2015). In regard to the definition and classification of celebrity social responsibilities, the literature primarily addresses ethical and legal issues, and has not formed a complete system index to address social responsibility. Therefore, this study has defined the concept of celebrity responsibility, and then works on creating a cinematic celebrities’ social responsibility evaluation system, which is key to achieving our research objectives.

### The relationship between social responsibility and public recognition of cinematic celebrities

Historically psychoanalytic theory indicates that recognition is the key mechanism for identity generation (Freud, 1921; Shimul et al., 2020). The study of the “star system” suggests that difference between stars and the public brings glamor and a “thrill” which produces that recognition. In this process of cultural consumption, it is easy to produce uncritical recognition (Stacey, 1991). There is close link between social acceptance and compliance with community rules, which is reflected in the data. The acquired ability of social acceptance for celebrities is dependent on their compliance with group rules. When an individual’s actions are consistent with community rules, the celebrity is able to acquire social recognition (Zhong and Li, 2016). When an individual’s behavior violates those group rules, they will be criticized by society (Ruan et al., 2005), and a celebrity’s misdemeanors then come to have a negative influence on the public’s liking of that celebrity.

Many academic studies focus on celebrity endorsement as one dimension of their responsibilities, and they have confirmed that there is a correlation between it and consumer recognition of the products the celebrities endorse. Compared with lack of such an endorsement, celebrity and online star endorsement have positive effects on consumers’ purchase intentions (Shi et al., 2021). However, negative celebrity publicity influences consumer attitudes and moral reputation plays an important role in that (Zhou and Whitla, 2013). The relationship between the celebrity and the consumer can also be negatively affected by a celebrity’s misbehavior (Banister and Cocker, 2014). Existing research analysis pay more attention to the relationship between the celebrity and the consumer from the market perspective (Pradhan et al., 2016; Sääksjärvi et al., 2016; Branchik and Chowdhury, 2017), but consumers are just part of the public who consume the image and activities of the celebrity (Littler, 2011) and they cannot as a group represent all the public who know and follow a particular celebrity (or celebrities).

In summary, there are few clearly delineated studies on the relationship between celebrity social responsibility and public recognition. To address this research gap, this study selects 286 movie stars as its research object, evaluates their social responsibility performances quantitatively, investigates public recognition, and explores the relationship between their social responsibility and their public recognition. It contributes to making clear the relationship between their social responsibility and public recognition as being an important and influential part of society.

## Research methodology

### Concept definition and hypothesis

Based on the above literature review, and considering the purposes of this paper, we define the social responsibility of cinematic celebrities as a requirement of society for that specific group, and one that is reflected in the expectations of the public and society. On the basis of the classification of CSR (Classon and Dahlstrõm, 2006; Marakova et al., 2021), combining the characteristics of individuals, celebrities’ social responsibility is divided into five categories: legal responsibility, professional responsibility, family ethical responsibility, public moral responsibility, and charity responsibility.

Public recognition refers to the degree of acceptance of a particular star from the public domain based on the understanding and judgment of the star’s social responsibility-related behaviors. As human beings, we exercise moral judgment: when we see an action or hear a story, we have an instant feeling of approval or disapproval (Greene and Haidt, 2002). The public can choose to approve or disapprove of particular celebrities according to their behaviors. A celebrity endorser enjoys public recognition and uses this recognition (McCracken, 1989), but morality is important for them to get and maintain that recognition. Celebrity endorsers’ credibility has positive impacts on consumer’s recognition of them and of the products they endorse (Pornpitakpan, 2008). Once a celebrity commits a transgression, consumer attitudes toward that celebrity become weaker (Shimul et al., 2020). Unethical behaviors exert a negative influence on both the celebrity and the brands they are endorsing (Thomas and Fowler, 2016). Therefore, the public always give more recognition to those celebrities who hold to their responsibilities. Based on the above discussion, we hypothesize:

H: Social responsibility performance of cinematic celebrities has positive impacts on their public recognition: specifically in the case of legal liability, professional liability, family ethical responsibility, public responsibility, and charitable moral responsibility, each of these five performances have positive impacts on public recognition.

### Research design

The research design includes: constructing the cinematic celebrities’ social responsibility evaluation system and determining the weights of the first-level and the second-level indexes; collecting the social responsibility performance data of the sample through content analysis taken from various forms of text; conducting a questionnaire design and investigation on the degree of public recognition; obtaining public recognition scores; and studying the relationship between responsibility and recognition through statistical methods.

In this paper, we applied the “Bass Law” to design the assessment scale. Bass “quantifies” the “level scale determined by the behaviors” (Bradburn et al., 2004). First, we developed an ideal behavioral scale by confirmation, definition and extraction of the star case samples. Second, we invited experts (e.g., from academia and journalism) to correct the work, and repeated this until most experts were in agreement with the scale. Finally, we generated and provided the scores of the behavior scale in accordance with the specific details, including degree, frequency, efficiency, scale, and other specified criteria.

#### Create celebrity social responsibility evaluation index system

Based on various media reports and online disclosures, this paper utilizes extracted data from 400 cases indicating social responsibility performance, and summarizes the primary irresponsible behaviors of the stars. Combining the definition and classifications of cinematic celebrity social responsibility, the construction of the evaluation system was undertaken.

Then, we randomly selected 30 movie stars for an evaluation test and consulted experts from the film industry, social responsibility research experts and entertainment reporters to review the system. According to the results of the evaluation test and the experts’ opinions, some indicators were added or adjusted. Indicators developed from this process include five first-level indicators, 25 second-level indicators, and 37 third-level indicators. In order to make the research results more objective, we use the “entropy” method to determine the weights of the indexes. Results are presented in Table 1.

**TABLE 1:** Cinematic celebrity social responsibility scale and weights.

First-level indicators	First-level indicators’ weights	Second-level indicators	Second-level indicators’ weights	Third-level indicators or rating basis
Legal responsibility	0.1836	False endorsement behavior	0.2544	The times of false endorsements, the endangered products, the attitude after-effects
		Drug activity	0.0542	Drug abuse and other drug-related behaviors
		Traffic violation	0.1037	Drunken driving, traffic accidents and other traffic violations
		Soliciting a prostitute	0.1045	Behavioral severity
		Tax violation	0.0916	Event severity
		Fighting	0.0789	Participation and severity of the incident
		Triad behavior	0.1341	Participation or behavioral severity
		Manipulating stock market behavior	0.0578	Degree of participation or manipulation
		Gambling behavior	0.1208	Behavioral severity
Professional responsibility	0.2409	Professional level	0.3920	Poor performance
		Dedication level	0.2760	Not being punctual at work, always using substitute, etc.
		Quality of work	0.3320	Poor quality or plagiarism
Family ethical responsibility	0.2410	Marital infidelity	0.6676	Behavioral severity
		Domestic violence	0.1973	Behavioral severity
		Not honoring Parents	0.1351	Behavioral severity
Public moral responsibility	0.2561	Hype	0.1882	Hype times and hype behavior
		Language	0.1710	Number and degree of swearing
		Manner	0.2140	Uncivilized behavior such as smoking and spitting in public places
		Indecent photo or video	0.2164	Event and behavioral severity
		Show off	0.1487	Degree of behavior
		Discrimination	0.0284	Behavioral severity
		Unpatriotic words and deeds	0.0334	Behavioral severity
Charity responsibility	0.0785	Fraud	0.3341	Event severity
		Charity negative news	0.1917	Event severity
		Charitable motive	0.4742	Utilitarian charity behavior

#### Quantitative evaluation of celebrity social responsibility

Of more than 7,000 film actors in China, we filtered and eliminated the lesser-known actors from that population in view of their lack of visibility to the public. The filtering resulted in a well-known population of 2,000 celebrities. We then applied a random sample method to those remaining 2,000 actors, resulting in a sample of 400 celebrities. Following the administration of the pilot survey, some stars were again filtered out of the sample in accordance with the survey results. Subsequently, 286 of the most popular film celebrities were selected for the final sample.

Employing the content analysis method, celebrity social behavioral data were retrieved from media reports. Behaviors were chosen in accordance with specific celebrity conduct that has been listed as one of the three-level indicators of the corresponding evaluation system (see Table 1).

The behavioral scores were generated relative to the results of the media and public report search. In this process, the entropy weight method was used. This particular method is more objective in providing index weights in comparison to other methods. It can be used to standardize the index value under the condition that the distribution of the index is not uniform and the quantity metric units are different across variables.

In accordance with “Bass Law,” the scores are divided into two categories: the first is the degree of the behavior, where the values are “none = 4, slight = 3, average = 2, serious = 1, very serious = 0,” and the second category is the frequency of the behavior, where the values are “no = 2, occasionally = 1, frequent = 0.” Next in the process, and according to the specific characteristics of each behavior, the degree index and the frequency index are individually calculated to determine the values of the third-level indexes.

Then we use the entropy weight method through MATLAB software to determine the weights. First, we standardized the third-level index scores. We denoted the third-level index scores as X_1_,X_2_,X_3_……X_k_, and the “n-th” score of the “j-th” indexes were X_1j_, X_2j_, X_3j_……X_nj_. We entered the data into the MATLAB and used the entropy method to calculate and report the normalized index value, called Y_1_,Y_2_,Y_3_……Y_k_, and ${\text{Y}}_{\text{ij}}=\frac{{\text{X}}_{\text{ij}}-\min\left({\text{X}}_{\text{ij}}\right)}{\max\left({\text{X}}_{\text{ij}}\right)-\text{min}\,\left({\text{X}}_{\text{ij}}\right)}$(1≤i≤n). Second, we calculated each index’s comentropy. According to the definition of comentropy (using “Ej” to symbolize it), each index score’s comentropy${\text{E}}_{\text{j}}=-{\left[\ln\left(\text{n}\right)\right]}^{-1}\,\sum_{\text{i}=1}^{\text{n}}p_{\text{ij}}\,{\text{lnp}}_{\text{ij}}$; where ${\text{p}}_{\text{ij}}={\text{Y}}_{\text{ij}}/\sum_{\text{i}=1}^{\text{n}}Y_{\text{ij}}$, if p_ij_ = 0, then$\lim_{{\text{p}}_{\text{ij}}\to 0}p_{\text{ij}}\,{\text{lnp}}_{\text{ij}}$ = 0. Last, we calculated the weight of the second index. According to each third-index score’s Ej value and the formula ${\text{W}}_{\text{i}}=\frac{1-{\text{E}}_{\text{i}}}{\text{k}-\sum {\text{E}}_{\text{i}}}$(i = 1,2,3……k),W_I_, the weights of the second-level index were calculated.

We obtained the scores of the second-level index by their weights. The weights and scores of the first-level indexes were obtained by the same method, and finally the social responsibility score of each star was calculated.

#### Questionnaire on public recognition

The survey questionnaire method was adopted to determine public recognition of selected celebrities. This recognition questionnaire was distributed to all provinces in China except Tibet and Taiwan. Each respondent involved in the inquiry only needed to evaluate the recognition of 20 randomly selected actors from the final 286 most popular celebrities’ sample. The questionnaire design was based on a 7-point Likert-scale, where “Strongly recognize” = 7, “Recognize” = 6, “Somewhat recognize” = 5, “Neutral” = 4, “Do not recognize somewhat” = 3, “Do not recognize” = 2, and “Do not recognize strongly” = 1. The questionnaires that were filled out by each survey participant were randomly sampled to ensure the reliability of the survey results. After having eliminated invalid responses, the average scores were public recognition scores of cinematic celebrities in the sample.

The survey was implemented using three diverse delivery methods: in-person, e-mail, and an online survey platform. A total of 2,600 questionnaires was distributed, yielding 2,248 qualifying, valid surveys. The questionnaire valid rate was 86.46% and the effective evaluation of each celebrity has been drawn more than 120 times. According to the method of determining the sample size for the statistical sampling survey, the result is reliable so long as each celebrity has been drawn upon 50 times or more. Therefore, the survey results meet the requirements of the investigative methodology. Among the final valid questionnaires, the age distribution is as follows: 18–35 years old accounted for 80.87%, 36–45 years old accounted for 13.92%, and respondents 46 years old and above accounted for 5.21%. The educational background distribution is as follows: junior college and below accounted for 10.85%, university undergraduate accounted for 78.43%, and those with master and doctorate degrees accounted for 10.72%. Occupational distribution is as follows: 966 students accounted for 42.97%, 356 government organs or institutions personnel accounted for 15.84%, 768 general enterprise personnel accounted for 34.16%, 64 entrepreneurs accounted for 2.85%, and the remaining 94 people accounted for 4.18%.

## Results

### Descriptive statistical analysis

The Cronbach’s alpha value of 0.807 and KMO value of 0.971 passed the reliability and validity test. In order to understand the overall characteristics of social responsibility and public recognition, a descriptive statistical analysis was initially conducted. The detailed results are shown in Table 2.

**TABLE 2:** Descriptive statistical analysis of cinematic celebrities’ social responsibility.

		Minimum value	Maximum value	Mean	Standard deviation	Variance
Scores of celebrity social responsibility	Legal responsibility	4.4081	7.5752	7.3130	0.5496	0.302
	Professional responsibility	3.8494	7.6988	7.3356	0.7232	0.523
	Family responsibility	0.9446	4.0000	3.8230	0.5695	0.324
	Public moral responsibility	1.7836	4.7124	4.4165	0.5744	0.330
	Charity responsibility	1.6940	4.5496	3.9567	0.2671	0.071
	Social responsibility total score	16.3923	28.5360	26.8447	1.9529	3.814
Scores of public recognition	Public recognition	2.8800	6.3370	5.0779	0.6137	0.377
Valid N (list state)	286					

Descriptive statistical analysis shows the lowest total score of social responsibility is 16.39, the highest score is 28.54, and the average score is 26.84 across 286 celebrities. For the five categories of responsibility, the average score of legal responsibility is the highest, followed by professional responsibility, and charity responsibility is the lowest. This shows that film celebrities generally have a strong sense of law and it is not common to violate the law; most of them abide by professional ethics, relative to their other responsibilities. However, in terms of charitable endeavors, celebrities are not generally active.

### Correlation analysis

**FIGURE 1:**
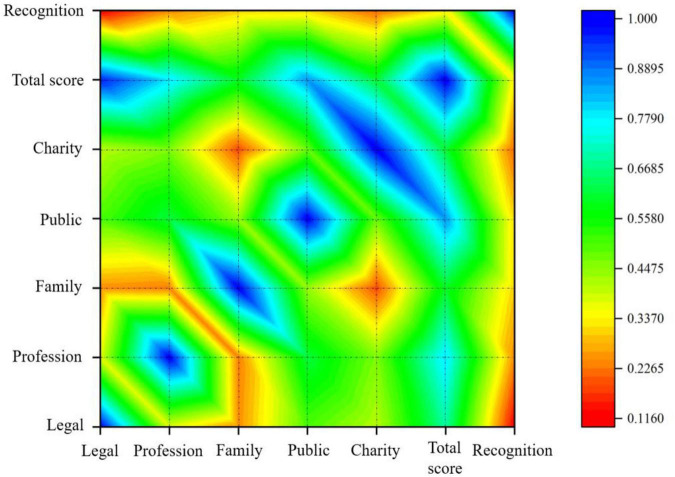
Visualized correlations.

Correlation analysis is designed to test whether or not there is correlation between variables. The Pearson correlation coefficient is commonly used. The results of the analysis are shown in Table 3. The table is presented in visual form in Figure 1.

**TABLE 3:** Correlations.

	Legal	Profession	Family	Public	Charity	Total score	Recognition
Legal	1	0.370**	0.248**	0.502**	0.407**	0.694**	0.118*
Profession	0.370**	1	0.236**	0.621**	0.460**	0.789**	0.245**
Family	0.248**	0.236**	1	0.436**	0.191**	0.603**	0.303**
Public	0.502**	0.621**	0.436**	1	0.475**	0.857**	0.312**
Charity	0.407**	0.460**	0.191**	0.475**	1	0.617**	0.212**
Total score	0.694**	0.789**	0.603**	0.857**	0.617**	1	0.333**
Recognition	0.118*	0.245**	0.303**	0.312**	0.212**	0.333**	1

**Correlation is significant at the 0.01 level (2-tailed). *Correlation is significant at the 0.05 level (2-tailed).

According to the analytical results, there is a statistically significant correlation between the overall performance of celebrity social responsibility and their public recognition scores. Specifically, legal performance is significantly and positively related to public recognition, such as in cases of drug abuse, traffic violations, false endorsements, and other responsibilities related to breaking the law. At the professional level, there is a statistically significant and positive correlation with recognition, such as professionalism and quality of work. There is also a significant positive correlation between the performance of celebrities within the family, such as derailment behavior, domestic violence and other types of family responsibility performance and public recognition. Finally, there is a significant positive correlation between the performance of public moral responsibility and recognition, as well as their performance in charity and motivation.

### Regression analysis

Before conducting the regression, we need to classify the scores for public recognition. Because public recognition score is based on a 7-point Likert Scale, the score of every celebrity is between 1 and 7. The classification criteria are: less than or equal to 1.5 = 1, greater than 1.5 and less than or equal to 2.5 = 2; greater than 2.5 and less than or equal to 3.5 = 3; greater than 3.5 and less than or equal to 4.5 = 4; greater than 4.5 and less than or equal to 5.5 = 5; greater than 5.5 and less than or equal to 6.5 = 6; more than 6.5 and less than or equal to 7 = s 7. Since the dependent variable is ordered classified data, while the independent variable is continuous data, the Ordinal Regression method is adopted.

#### Regression analysis of the total score of social responsibility of cinematic celebrities and public recognition

The results are shown in Tables 4–6. The parallel test result in Table 4 shows that the *P*-value is 0.618, which is greater than 0.05, indicating that the model accepts the null hypothesis, that is, the parallel test is satisfied, and further analysis can be performed. Table 5 shows the likelihood ratio test results, demonstrating the overall effectiveness of the model. The *P*-value is less than 0.001, illustrating the overall model is meaningful, because at least one variable coefficient does not equal 0.

**TABLE 4:** Test of parallel lines.

Model	−2 Log Likelihood	Chi-Square	Df	Sig.
Null hypothesis	278.627			
General	277.665	0.961	2	0.618

The null hypothesis states that the location parameters (slope coefficients) are the same across response categories.

Link function: Logit.

**TABLE 5:** Model fitting information.

Model	−2 Log Likelihood	Chi-Square	Df	Sig.
Intercept Only	300.729			
Final	278.627	22.102	1	0.000

Link function: Logit.

**TABLE 6:** Parameter estimates.

		Estimate	Std. Error	Wald	Df	Sig.	95% confidence interval	
							
							Lower bound	Upper bound
Threshold	(Recognition Classification = 3)	3.582	1.645	4.743	1	0.029	0.358	6.806
	(Recognition Classification = 4)	6.024	1.645	13.411	1	0.000	2.800	9.249
	(Recognition Classification = 5)	9.101	1.704	28.531	1	0.000	5.762	12.441
Location	Total score	0.293	0.062	21.966	1	0.000	0.170	0.415

Cox and Snell R-Square: 0.074.

Nagelkerke R-Square: 0.087.

McFadden R-Square: 0.039.

The summary of the model results is shown in Table 6. The pseudo-R-squared value of the model is 0.074, meaning that the total score of social responsibility can explain the reason for the change of 7.4% of public recognition. The regression coefficient value is 0.293, with a significance level of 0.01, which means that the total level of social responsibility has a significant positive impact on public recognition.

#### Regression analysis of the performance of various dimensions of social responsibility of cinematic celebrities and public recognition

The regression analysis method is the same as above. The results are shown in Tables 7–9. The parallel test results of the model in Table 7 show that the *P*-value is 0.494, which is greater than 0.05, indicating that the model accepts the null hypothesis, that is, the parallel test is satisfied, and further analysis can be performed. Table 8 illustrates the results of the model likelihood ratio test, and because *P* < 0.001 the model as a whole is meaningful.

**TABLE 7:** Test of parallel lines*^a^*.

Model	−2 Log Likelihood	Chi-Square	Df	Sig.
Null hypothesis	263.881			
General	254.471	9.410	10	0.494

The null hypothesis states that the location parameters (slope coefficients) are the same across response categories.

^a^Link function: Logit.

**TABLE 8:** Model fitting information.

Model	−2 Log Likelihood	Chi-Square	Df	Sig.
Intercept Only	300.729			
Final	263.881	36.848	5	0.000

Link function: Logit.

**TABLE 9:** Parameter estimates.

		Estimate	Std. Error	Wald	Df	Sig.	95% CI	
						
							Lower bound	Upper bound
Threshold	(Recognition classification = 3)	0.256	2.099	0.015	1	0.903	−3.858	4.371
	(Recognition classification = 4)	2.881	2.106	1.871	1	0.171	−1.247	7.009
	(Recognition classification = 5)	6.056	2.128	8.098	1	0.004	1.885	10.228
Location	Legal	−0.584	0.259	5.082	1	0.024	−1.092	−0.076
	Career	0.343	0.217	2.511	1	0.113	−0.081	0.768
	Family	0.562	0.234	5.757	1	0.016	0.103	1.021
	Public	0.794	0.313	6.424	1	0.011	0.180	1.408
	Charitable	0.219	0.528	0.172	1	0.678	−0.816	1.255

Cox and Snell R-Square: 0.121.

Nagelkerke R and lt-Square: 0.141.

McFadden R-Square: 0.066.

Link function: Logit.

The summary of the parameter estimate results is shown in Table 9. The pseudo-R-squared value of the model is 0.121, meaning that legal liability, professional responsibility, family responsibility, public responsibility, and charity responsibility can explain the reason for the change in public acceptance of 12.1%. The regression coefficient of legal liability is −0.584, and the significance level is 0.05, indicating that legal responsibility has a significant negative impact on public recognition. In contrast, the correlation coefficients for family responsibilities and public responsibilities are 0.562 and 0.794, respectively, meaning that these two responsibilities have a significant positive impact on public recognition.

## Discussion

Based on the empirical evidence provided here, it is clear that the better the overall performance of social responsibility by celebrities in China, the higher their public recognition scores. In particular, the greater their presentation of family and public responsibility, the higher their public recognition scores. Professional and charitable responsibilities do not enter into the final regression equation, which indicates that even when stars lack sense and behaviors in their professional or charitable responsibility, their public recognition scores will not be significantly impacted.

As demonstrated in Table 9, celebrity legal responsibility is negatively correlated with public recognition. There may be several reasons for this. First, due to information asymmetry between public figures and the public, stars’ illegal behavior can become less visible. Second, some people, especially young people, have to some extent irrational attitudes toward celebrities (and in this study the questionnaire respondents of public recognition are indeed mainly young people). Because of irrational “fanaticism” (or fan behavior), they do not care if celebrities break laws (Guo, 2015). Third, celebrity violations are generally minor offenses, making it easier to obtain public forgiveness.

Both public moral responsibility and family ethical responsibility have positive impacts on public recognition, which indicates that the public takes a stronger interest in celebrities’ public morality and family ethical performance. Public behavior is more likely to be exposed by the media and to impact on public recognition. In the same way, the family and private lives of cinematic celebrities have always been hot topics for the public and the media. The media and the public have a relatively consistent evaluation of celebrities’ behaviors, that is, those who are engaged in divorce and/or family disorder are less likely to gain positive recognition.

Professional responsibility of cinematic celebrities has no statistically significant influence on their public recognition. The data demonstrate that the public pays little attention to professional qualities. Similarly, charitable responsibility has no significant influence on public recognition for popular actors, one of possible reasons being that celebrities’ acts of charity are dismissed as simply serving their self-branding.

## Conclusion

### Conclusions and recommendations

We conclude this study with the following three main points. First, film celebrities should be encouraged to establish a consciousness about their social responsibility. According to the qualitative and quantitative analysis in this research, the overall social responsibility of film stars has a significant positive impact on their public recognition, indicating that society as a whole has an objective understanding and positive expectation of celebrities. These results are consistent with previous similar marketing based studies (Malodia et al., 2017; Alharbi et al., 2022). They argued that the evaluation could also be linked to consumers’ ethnicity and the use of cultural symbols. As being a high-income group providing cultural products for their society, celebrities should constantly improve their moral level and give back to their society, which is, after all, also the foundation of their sustainable development.

Secondly, celebrities are encouraged to pay special attention to their influence on public morality. This research shows that public moral and family ethical responsibility have a significant impact on the public recognition. As public figures, stars have an obligation to guide the healthy development of society, and they should strengthen their self-cultivation in public moral behavior. Family is the basic unit of society, and responsibility to the family is thus very important, and contributes to the overall health of and cohesion within and across communities. Celebrities should be responsible for their families, and in particular seek to avoid violating norms of family morality through taking part in social responsibilities. These results are positively contradict with recent study. Huang argued that public figures for endorsement of commercial advertising ruins their good public images. Once the commercial advertisements or the products are illegal or the companies involved in them are known to have committed illegal behavior, the public image of public figures will be greatly affected. Their reputation and credibility would be questioned by the public. This effect will be transferred to the public service advertisement that the figure endorsed. At this time, their opinions are not convincing at all in the public service advertising. The public figures that lose credit would bring disgrace on their own head. In contrast our study support suggest that through adopting social responsibilities, they could maintain their better image in society forever.

Lastly, the public’s cognitive evaluation needs to become more rational. As confirmed in the research results, professional and charitable responsibility did not enter into the regression analysis equation, indicating the performance of film stars in those two categories will not significantly impact public recognition statistically. These results reflect a kind of “star chaser” phenomenon and other non-rational psychological responses among the public when evaluating movie star popularity. In particular, more than 80% of questionnaire respondents were young people, so that the results reveal a degree of unthinking pursuit of and interest in stars among the younger generation in China. Thus, it can be argued that the public’s evaluation of movie stars is not always morally rational or objective (Duan, 2007), and we suggest that the level of cognition and evaluative methods can be improved. Society itself should also guide young people to pursue their interest in and approval of movie stars more rationally (Lv and Zhang, 2019).

### Limitations

The investigation of cinematic celebrity social responsibility was based on information and data collected from media reports. Yet there remains a possibility that media reports and online comments are inconsistent with the reality. Due to the variation within and across film star groups, it is difficult to verify social responsibility behaviors one by one, so that there are considerable limitations inherent in these media sources of data.

Due to limited financial resources, in contrast to demanding research costs, the survey of public recognition was distributed to only 2,600 questionnaire respondents. Although the number is adequate for research theoretically and statistically for the 286 sample size in the study, the results would have been more scientific if the questionnaire had been distributed to a broader and more comprehensive group of the public.

In addition, this particular survey scale was designed to capture and measure irresponsible celebrity social behavior by using a behavioral model. Due to the limited availability of the data, there is still room for correcting the scale.

### Future directions

Future research might define and evaluate social responsibility in a more rigorous and objective way, including utilizing broad data collection and analytical methods. Administering a survey questionnaire designed to measure more positive social responsibility behaviors is recommended. Future research may also focus on the reasons for the differences in social responsibility consciousness and behavior and discover the main influencing factors. In addition, from the perspective of the public, researching the objective degree of public evaluation is also a research direction.

## Data availability statement

The raw data supporting the conclusions of this article will be made available by the authors, without undue reservation.

## Ethics statement

Ethical review and approval was not required for the study on human participants in accordance with the local legislation and institutional requirements. Written informed consent from the patients/participants or patients/participants legal guardian/next of kin was not required to participate in this study in accordance with the national legislation and the institutional requirements.

## Author contributions

All authors listed have made a substantial, direct, and intellectual contribution to the work, and approved it for publication.
